# An NMR Study of Biomimetic Fluorapatite – Gelatine Mesocrystals

**DOI:** 10.1038/srep15797

**Published:** 2015-10-30

**Authors:** Anastasia Vyalikh, Paul Simon, Elena Rosseeva, Jana Buder, Ulrich Scheler, Rüdiger Kniep

**Affiliations:** 1 Institut für Experimentelle Physik, TU Bergakademie Freiberg, Leipziger Straße 23, 09596 Freiberg, Germany; 2 Leibniz-Institut für Polymerforschung Dresden e.V., Hohe Str. 6, 01069 Dresden, Germany; 3 Max-Planck-Institut für Chemische Physik fester Stoffe, Nöthnitzer Str. 40, 01187 Dresden, Germany; 4 University of Konstanz, Physical Chemistry, POB 714, D-78457 Konstanz, Germany

## Abstract

The mesocrystal system fluoroapatite—gelatine grown by double-diffusion is characterized by hierarchical composite structure on a mesoscale. In the present work we apply solid state NMR to characterize its structure on the molecular level and provide a link between the structural organisation on the mesoscale and atomistic computer simulations. Thus, we find that the individual nanocrystals are composed of crystalline fluorapatite domains covered by a thin boundary apatite-like layer. The latter is in contact with an amorphous layer, which fills the interparticle space. The amorphous layer is comprised of the organic matrix impregnated by isolated phosphate groups, Ca_3_F motifs and water molecules. Our NMR data provide clear evidence for the existence of precursor complexes in the gelatine phase, which were not involved in the formation of apatite crystals, proving hence theoretical predictions on the structural pre-treatment of gelatine by ion impregnation. The interfacial interactions, which may be described as the glue holding the composite materials together, comprise hydrogen bond interactions with the apatite PO_4_^3−^ groups. The reported results are in a good agreement with molecular dynamics simulations, which address the mechanisms of a growth control by collagen fibers, and with experimental observations of an amorphous cover layer in biominerals.

Mesocrystals or *mesoscopically structured crystals* are highly sophisticated materials, which are built up from individual nanocrystals as building blocks and which are aligned in a crystallographically ordered way^1^. Four principal possibilities are suggested for the remarkably ordered alignment and assembly of nanoparticles in mesocrystals^2^. These are an oriented organic matrix, the presence of electrostatic or magnetic fields, an epitaxial growth of nanoparticles with a mineral bridge between them and, finally, alignment by spatial constraints. Mesocrystals are much more common than assumed so far, but it is difficult to detect them, as they usually scatter X-rays like a single crystal and appear well-facetted^3^. Their unique controlled structure supports a unifying crystallization scenario, which combines both atomic/molecule-mediated (classical) and particle-mediated (non-classical) pathways^4^. Due to their unique combination of structural features related to high crystallinity and high porosity, as well as nanoparticles properties, mesocrystals provide solutions in many application areas. Recent progress on mesocrystals and their potential applications as heterogeneous photocatalysts, electrodes, optoelectronics, biomedical materials, hard templates, sensors and lightweight structural materials have been extensively reviewed in recent years^5^,^6^,^7^,^8^,^9^,^10^. Moreover, the possibility to tune the particle dimensions and the interactions between the physical and physicochemical properties of the individual nanoparticles by governing reaction conditions offers potential for developing novel materials with tailored functionalities. A natural process of biomineralization has also been found to take place via the particle-mediated pathway of mesocrystallization^11^,^12^. Since biominerals combine complex morphology and unique functional properties as the result of evolution-optimized processes, comprehensive insights into the biomineralization mechanisms open up promising approaches for bioinspired and biomimetic materials design^11^,^13^. In addition, adopting the bioinspired approach enables not only production of novel functional materials, but also better understanding of biomeralization processes through controlled synthesis and the *in situ* characterization methods. Recently, using a combination of *in-situ* methods the biomimetic nucleation of calcium phosphate, which is a major inorganic constituent of the hard tissues of animals, has been shown to occur through a unifying crystallization process^14^. Practical application of biomimetic strategies has been recently demonstrated for remineralization of human dentine^15^ and *in vitro* repair of a seashell^16^. It was suggested that organic macromolecules play important roles in the repair process and can control the mineralization reactions on several levels, including the formation of prenucleation clusters. In a recent review, Cölfen described the polymers that are useful for this purpose and the experimental conditions suitable for directing a crystallization reaction in the desired direction^17^.

However, a classical epitaxy paradigm, e.g., an epitaxial match between the structural organic matrix and the mineral, has been disproven by a HRTEM study of synthetic aragonite and of *Haliotis laevigata* gastropod nacre^18^,^19^. The existence of the amorphous layer around aragonite platelets in nacre is explained by the exclusion of impurities and expulsion of macromolecules throughout the crystallization, which prevents further crystallization. It has been suggested that this layer could also provide better adhesion and mechanical performance of the hybrid material. In our previous studies of synthetic hydroxy- and fluorapatite mesocrystals a disordered (amorphous) layer, which covers the apatite crystalline domains and is coordinated to water and the organic matrix, has been identified by solid-state NMR^20^,^21^. Thus, a disordered layer between aligned single crystalline nanoparticles embedded in an amorphous organic phase therefore seems to be an intriguing hypothesis for biominerals, which demonstrate mesocrystalline properties. Despite its relevance, little is known about the constituents and the molecular-level structure of this composite material. Therefore in the present work we continue our efforts on the structural elucidation of biomimetic fluoroapatite—gelatine nanocomposites.

The mesocrystal system fluoroapatite—gelatine grown by double-diffusion is a fair illustration of self-organized morphogenesis and a hierarchical structure on the mesoscale^22^,^23^,^24^,^25^,^26^,^27^,^28^. Growth of the nanocomposites starts with an elongated hexagonal prismatic seed (5–20 μm in length, Fig. 1a), followed by a self-similar branching of dumbbell states (Fig. 1b,c), which finally leads to the development of closed spheres. As proven by electron holography the fractal growth mechanism, which results in the dumbbell-like nanocomposite aggregates, is controlled by the intrinsic electrical dipole fields induced by an aligned polar biomolecule^26^,^29^. The inner architecture of the young seed, as indicated by high-resolution TEM, is built by a parallel stacking of elongated subunits oriented with their long [001] axes parallel to the seed. X-ray diffraction has revealed the presence of crystalline fluorapatite within the hexagonal prismatic seed, while a tilted mounting of the later crystal generations during the fractal morphogenesis has been observed^30^. On the mesoscopic scale, the so-called mosaic arrangement is suggested, where the periodic mineral domains of a nanocomposite subunit grow around a central protein triple-helix^27^. It has been shown that the domains do not perfectly match, giving rise to a healing layer at the outer borders to match the hexagonal pattern. As evidenced by electron microscopy, the gelatine molecules are surrounded by assemblies of fluorapatite nanoparticles arranged in a honeycomb-like network providing homogeneous intergrowth of the inorganic and organic components. Using solid state NMR we studied the interfacial mineral-organic structure in large spherical aggregates up to 100 μm in size, which represent the final growth state of the fractal-grown fluorapatite-gelatin composites^20^,^21^. As it is obvious that the fundamental principles of mineral growth, passivation and stabilization are already included in the early stages of composite growth, in the present work we focus on the study of the hexagonal prismatic seeds and dumbbells, thus the initial states of growth of fluorapatite/gelatin composites.

## Experimental section

### Samples

Details of synthesis of fluorine-gelatine nanocomposites using the double diffusion technique have been published previously^24^,^30^,^31^. A composite aggregate extracted at the early growth stage was ground, washed 3 times for 20 min in distilled water at 40 °C, then centrifuged and finally dried at 40 °C in order to remove a fraction of gelatine, which is only physisorbed on the aggregates’ surface and not integrated in the composite.

### X-Ray diffraction

X-ray powder data were collected in transmission mode using a Huber G670 Image Plate Camera, Cu K_α1_ radiation (*λ* = 1.540598 Å) and germanium (111) monochromator. Lattice constants *a* and *c* of apatite were calculated by least-squares refinements using LaB_6_ (cubic, *a* = 4.15692 Å) as internal standard. The measurements were performed before and after heating to 250 °C to study the effect of release of hydroxyl groups and water from the crystal structure.

### SEM

The morphology was studied by scanning electron microscopy (SEM). SEM investigations were performed by means of a Philips ESEM Quanta 200FEGI system operated in high-vacuum mode at an acceleration voltage of 25 kV (FEI, Eindhoven, NL). For investigation under high vacuum, the samples were coated by a thin gold layer (for 30 seconds), and secondary electron images were recorded.

### NMR

For NMR experiments the samples were filled into zirconia rotors and closed tightly. ^1^H and ^19^F NMR experiments were carried out on a (11.7 T) Bruker Avance III 500 spectrometer operating at resonance frequencies of 500.1 MHz for ^1^H and 470 MHz for ^19^F using a 2.5 mm HFX-MAS probehead with a spinning frequency of 30 kHz. For ^1^H MAS NMR the 90°-pulse duration of 3 μs, a recycle delay of 5 s and high-power ^19^F TPPM (50 kHz) decoupling were applied. The ^19^F MAS NMR experiments were acquired with a 90°-pulse duration of 4.5 μs, a recycle delay of 40 s, a number of repetitions of 64 and TPPM (50 kHz) decoupling on protons. For the ^19^F{^1^H} cross polarization (CP) measurements contact times in the range of 100 μs to 1500 μs and a recycle delay of 5 s were used. The two-dimensional ^19^F-^1^H HETCOR experiment was performed at 30 kHz MAS and a contact time of 500 μs. A recycle delay of 5 s and 512 scans per t_1_ time increment were applied. A total of 32 t_1_ slices with a 50 μs time increment in the indirect dimension were acquired.

^31^P NMR spectra were acquired on a (7 T) Bruker Avance 300 spectrometer operating at resonance frequencies of 300.1 MHz for ^1^H and 121.5 MHz for ^31^P employing a BL4 HX 4 mm MAS probehead. The ^31^P MAS NMR experiments were performed at spinning frequencies of 10 and 14 kHz with either ^1^H or ^19^F high-power TPPM decoupling, a 90°-pulse duration of 4.5 μs, a recycle delay of 30 s and 16 repetitions. For ^31^P{^1^H} CP measurements a recycle delay of 5 s and a contact time varied from 100 μs to 4 ms were used. Two-dimensional ^31^P-^1^H HETCOR experiments were performed with a spinning speed of 10 kHz and contact times of 1.5 ms and 3 ms. A recycle delay of 5 s, 32 scans per t_1_ time increment and a 75.53 μs time increment in the indirect dimension were used. ^31^P{^19^F} CP MAS (10 kHz) spectra were measured at a contact time in the range of 0.1 ms to 4 ms, 16 repetitions and a recycle delay of 50 s.The ^33^P-^19^F HETCOR spectrum (10 kHz MAS) was acquired at a contact time of 1.5 ms, 64 scans per t_1_ time increment and a 200 μs time increment in the indirect dimension. All ^1^H chemical shifts were referenced to tetramethylsilane (TMS) at 0 ppm using poly(vinylidene fluoride) as an external reference (δ^1^H = 2.9 ppm); ^19^F chemical shifts were referenced relative to CFCl_3_ at 0 ppm using PTFE as an external reference (δ^19^F = −122 ppm); powdered ammonium dihydrogen phosphate was used to reference the ^31^P spectra at 0.72 ppm relative to 85% phosphoric acid. All spectra were fitted using Dmfit^32^.

## Results

### SEM

**Figure 1:**
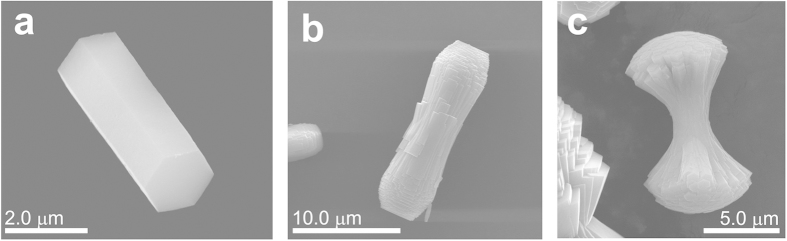
SEM images of fluorapatite-gelatine nanocomposite aggregates including (**a**) an elongated hexagonal-prismatic seed, (**b**) a dumbbell with initial splitting and (**c**) a “large” dumbbell with fractal branching.

SEM images of the fluorapatite-gelatine composite demonstrate that the sample under study represents a combination of prismatic seeds (Fig. 1a) and dumbbell-like aggregates (Fig. 1b,c). According to the previously published results, this corresponds to earlier stages of morphogenesis, when an initially formed elongated hexagonal prismatic seed starts to grow and split, demonstrating fractal morphogenesis.

### ^19^F NMR

**Figure 2:**
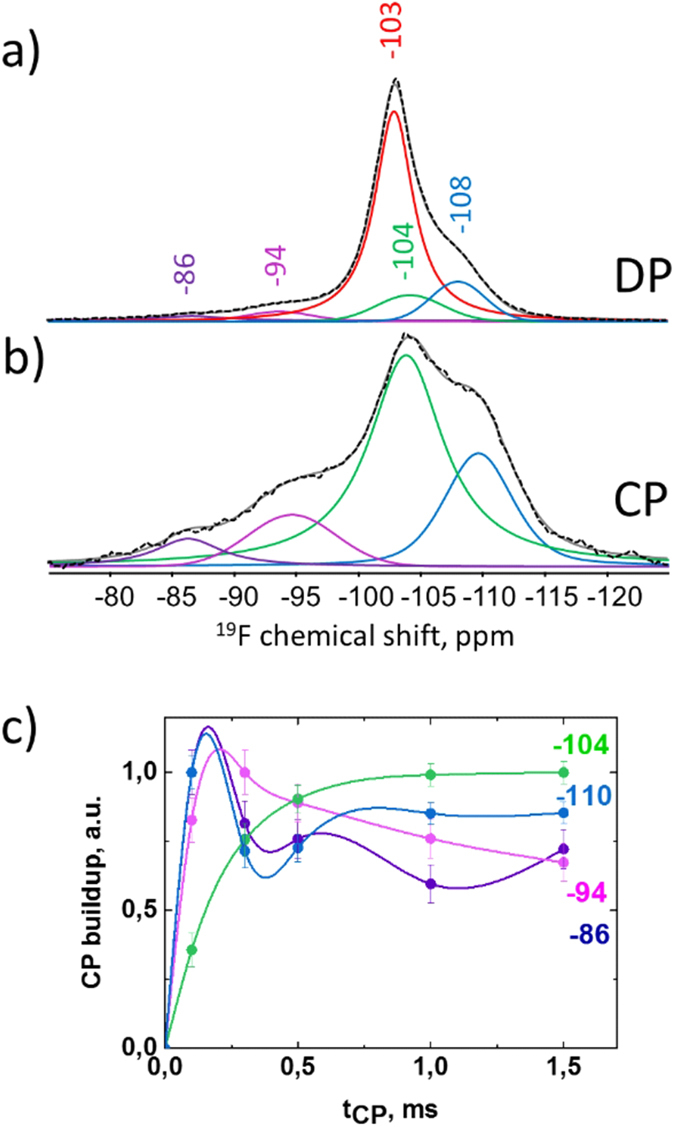
^19^F MAS NMR spectra detected using (**a**) direct polarization and (**b**) cross-polarization (CP) from ^1^H together with the deconvolution results.Spectra are normalized to a maximum intensity. (**c**) CP build-up as a function of the contact time t_CP_ for four spectral components. Connecting lines are introduced for visualization.

In the ^19^F MAS NMR spectrum of nanocomposite recorded by direct polarization (DP), five spectral components are identified (Fig. 2a), whose fit parameters are summarized in Table 1. The chemical shift values δ_iso_^19^F for four lines are close to those found for the spherical aggregates reported in ref. 21. In the present study a minor high-field shoulder centred at ca. δ_iso_^19^F = −86 ppm has been found in addition. Distribution of the component intensities essentially changes in the spectrum obtained by the cross-polarization (CP) showing different ^1^H-^19^F proximities of corresponding species (Fig. 2b). The peak at δ^19^F = −103 ppm is no longer visible in Fig. 2b, which indicates lack of spatial correlation to the protons and proves its assignment to pure crystalline fluorapatite^21^,^33^. Figure 2c shows the different CP build-up behaviour for the spectral components determined in the ^19^F{^1^H} CP MAS spectrum. In contrast to other signals, the ^19^F peak at −104 ppm continues growing up to t_CP_ = 1 ms. Such behavior is characteristic of the crystalline apatite structure. Moreover, its chemical shift, which is very close to the pure crystalline fluorapatite, can indicate that the local apatite structure around this fluorine site is preserved^34^. Faster CP build-up of the remaining ^19^F signals, which reach a maximum at t_CP_ = 0.1 ms (Fig. 2c), points to the close proximity to protons, perhaps, indicating covalent binding. In our previous work we attributed the ^19^F signal at ca. −109 ppm to an amorphous layer in close contact to the organic phase, while the signal around −96 ppm has been tentatively assigned to partial fluorination of gelatine during the formation of the nanocomposite^21^. Here we apply two-dimensional ^19^F-^1^H heteronuclear correlation (HETCOR) spectroscopy in order to analyse cross-correlation between the ^1^H and ^19^F signals and to get detailed information on the spatial association between ^1^H and ^19^F sites.

**Figure 3:**
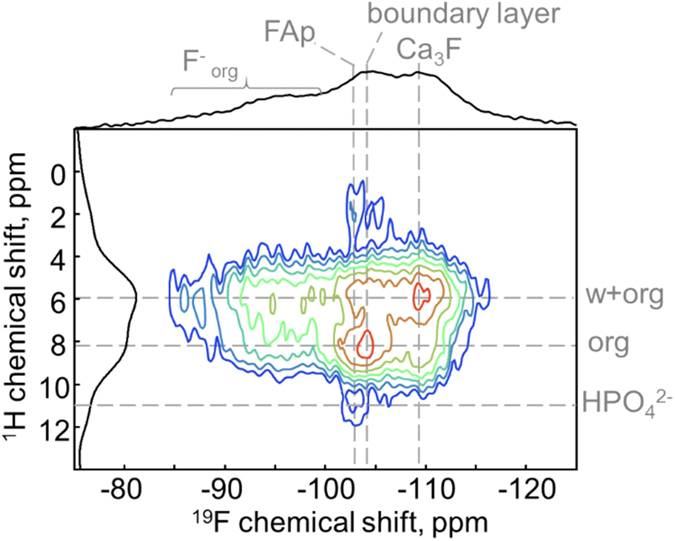
^19^F-^1^H HETCOR spectrum with the corresponding sum-up projections.

The ^19^F-^1^H HETCOR spectrum (Fig. 3) demonstrates strong correlation to the peaks at δ^1^H = 8.2 ppm and δ^1^H = 5.8 ppm, which are attributed to certain molecular fragments of gelatin and water, respectively. The low-field shift (to higher ppm-values) relative to bulk water (δ^1^H = 4.8 ppm) is explained by strong hydrogen bonding of the water molecules to the organic molecule or orthophosphate groups. The crystalline fluorapatite peak at δ^19^F = −103 ppm also appears in the HETCOR spectrum, indicating the minor cross-signals at δ^1^H = 2.0 ppm and δ^1^H = 11.4 ppm. The former arises from isolated water molecules in the apatite channels and has been observed in the final growth stage^21^. The latter is assigned to HPO_4_^2−^ groups, which are frequently observed in the spectra of hydroxyapatite^35^ and other relevant systems, such as biological hard calcified tissues^36^,^37^ and biomimetic calcium phosphates^21^. The signals in the range of δ^19^F = −86 to −100 ppm cross-correlate to the ^1^H peaks related to water and the organic matrix.

### ^31^P NMR

**Figure 4:**
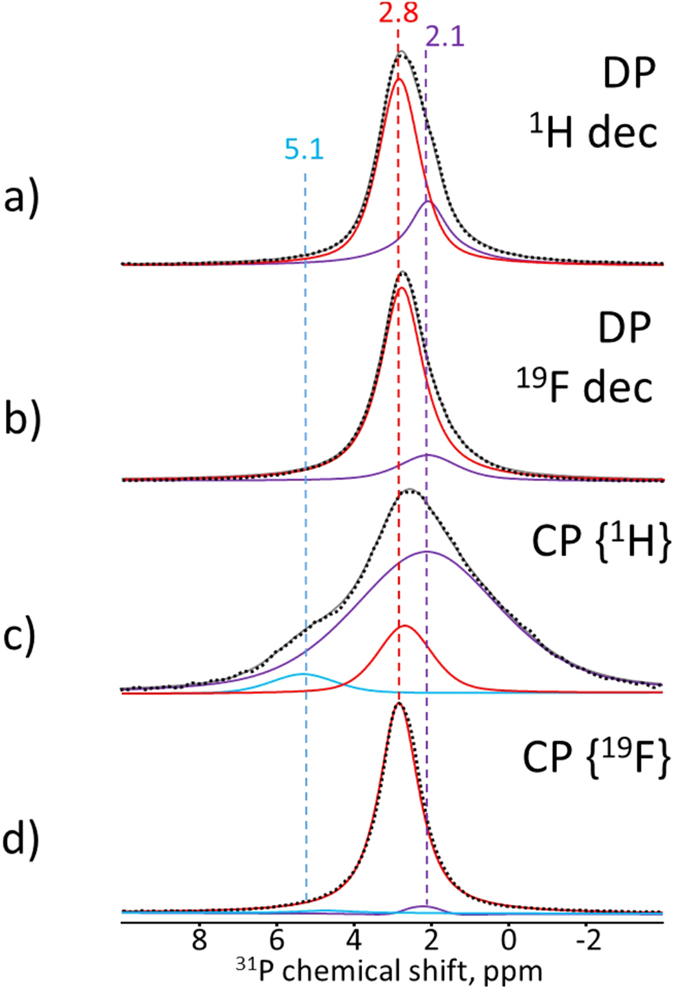
^31^P MAS (10 kHz) NMR spectra detected using (**a**) direct polarization with high-power ^1^H decoupling, (**b**) direct polarization with high-power ^19^F decoupling, (**c**) cross-polarization from ^1^H and (**d**) cross-polarization from ^19^F together with the deconvolution results. Spectra are normalized to a maximum intensity.

The ^31^P MAS spectra are presented in Fig. 4. In contrast to the ^31^P MAS NMR spectrum of the spherical aggregates^21^, which represents a single fluorapatite ^31^P signal at δ^31^P = 2.8 ppm, in the early stage sample, in addition, a second component at δ^31^P = 2.1 ppm appears (Fig. 4a,b). Applying heteronuclear high-power decoupling enables linewidth narrowing due to suppression of the corresponding heteronuclear dipolar interactions. The effect of two different decoupling channels on the ^31^P spectrum (^1^H- and ^19^F-decoupled, Fig. 4a,b, respectively) is evidently indicating that the ^31^P site which gives a signal at 2.1 ppm is surrounded by a large proton bath. Indeed, this component is essentially amplified in the ^31^P spectrum recorded by cross-polarisation from protons (Fig. 4c) and nearly disappears when measured by cross-polarisation from ^19^F (Fig. 4d). The ^31^P{^19^F} CP spectrum (Fig. 4d) proves that the peak at 2.8 ppm arises from a pure fluorapatite structure^21^,^23^,^24^. In addition, a very low fraction of a non-apatite origin is visible in the ^31^P{^1^H} CP spectrum at 5.1 ppm. We can thus conclude that besides the dominating contribution from fluorapatite at δ^31^P = 2.8 ppm in the ^31^P spectra, there are small fractions of phosphorus-containing species spatially associated to protons.

**Figure 5:**
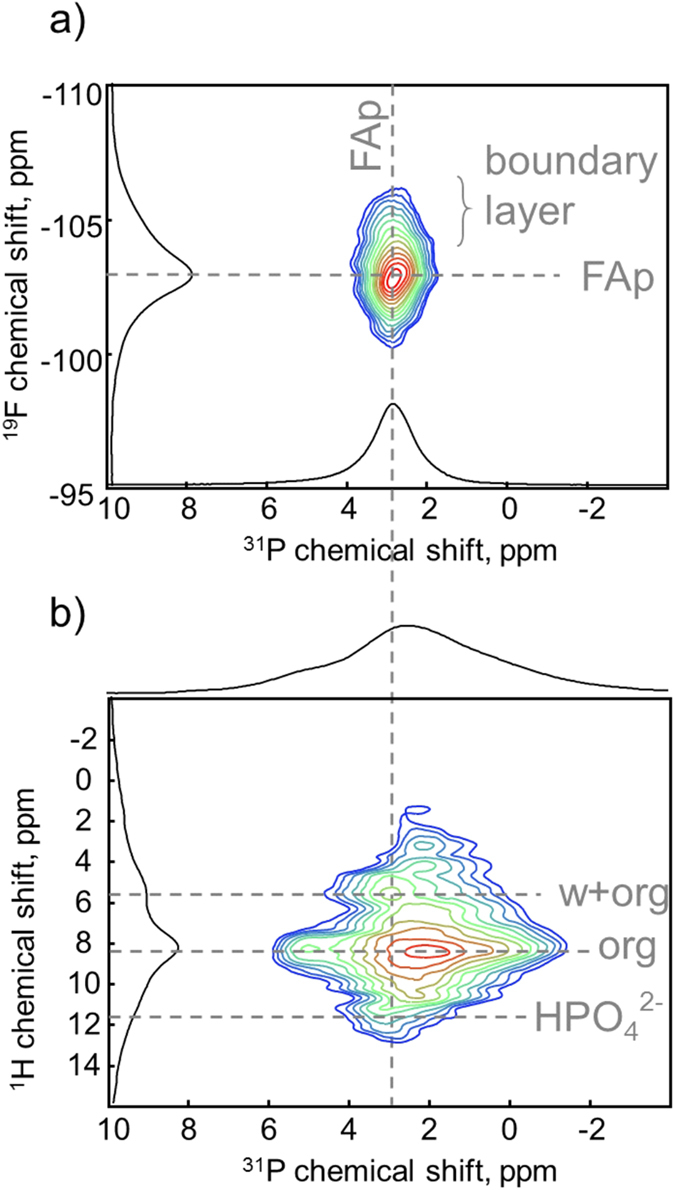
(**a**) ^31^P-^19^F HETCOR and (**b**) ^31^P-^1^H HETCOR spectra with the corresponding sum-up projections.

Two-dimensional HETCOR experiments, where ^31^P chemical shifts are correlated to either ^19^F or ^1^H in the indirect dimension enable us to get deeper insight into the structural environment around phosphorous atoms. The ^31^P-^19^F HETCOR spectrum (Fig. 5a) demonstrates a single asymmetric cross-peak with a maximum intensity at δ^31^P = 2.8 ppm and δ^19^F = −103 ppm. This is a strong indication of the pure fluorapatite structure and support for the assignment given above. In the ^19^F sum projection a low-field shoulder at ca. −104 ppm is observed, which hints to the presence of another component not well separated in the 2D spectrum. Actually, its ^19^F line position and correlation to the peak at δ^31^P = 2.8 ppm points to its apatite origin. It is noteworthy that no other ^31^P-^19^F cross-peak is observed, demonstrating that the other peaks listed in Table 1 are of non-mineral origin at this growth stage.

In contrast, ^31^P-^1^H HETCOR shows a more complex spectrum (Fig. 5b). First, the cross-peaks associated with the apatite structure (δ^31^P = 2.8 ppm) are visible at δ^1^H = 5.8 ppm and δ^1^H = 11.4 ppm (dashed vertical line in Fig. 5b). They prove the presence of surface water and hydrogenated phosphate groups, respectively. Similar correlation signals have been observed in synthetic biomimetic nanocomposites^20^, as well as in biominerals such as joint mineralized cartilage^38^, animal bone^39^,^40^, and rat dentine^41^. Further, two strong cross-signals appear at δ^31^P/δ^1^H of 2.1 ppm/8.4 ppm and 5.0 ppm/8.4 ppm (Fig. 5b). According to the literature these ^31^P signals can be assigned to orthophosphate groups located at the mineral surface^21^,^42^,^43^ or to the side products of a double-diffusion reaction such as NaH_2_PO_4_H_2_O, K_2_HPO_4_· 3H_2_O and NaNH_4_HPO_4_· 4H_2_O, whose ^31^P signals are known to appear at 2.3, 2.1 and 5.1 ppm, respectively^44^. The signal at δ^31^P = 5.1 ppm has been observed in the one-dimensional ^31^P{^1^H} CP NMR spectrum (Fig. 4c) and attributed to non-apatite species. In either case, it is evident that these signals can arise from phosphate groups of non-apatite origin interacting with organic matrix. Note, that athough a low fraction of apatite-channel water has been found in ^19^F-^1^H HETCOR NMR (Fig. 3), the correlation signals at δ^1^H = 0 ppm and δ^1^H = 1.5 ppm, characteristic of apatite OH^-^ groups and apatite channel water, respectively, are absent here.

## Discussion

Fitting and quantification of the ^19^F MAS NMR spectrum obtained by direct polarization provides information about the relative fraction of each species resolved in the spectra. This allows us to get insight into the structure of the internal and interfacial regions of the early stage nanocomposite aggregates.

As suggested in our previous work for the final growth stage the mineral component is composed of crystalline apatite-like core surrounded by a disordered layer with first motifs of the apatite structure, which interacts with organic matrix^21^. In the earlier stage sample, the dominating contribution (72%) from all fluorine-containing species comes from crystalline fluorapatite (FAp) (Table 1, Fig. 2a). The 2D data (Fig. 3) show that FAp is associated weakly to HPO_4_^2-^ groups and isolated water molecules. The latter can be entrapped as structural defects and substitute fluorine ions in the apatite channels. The cross-peaks between FAp and gelatine/water molecules, which would prove the scenario of the periodic FAp domains’ growth around a protein triple-helix^27^,^31^, could be hidden beneath the large peaks due to their low fraction.

In the following, the origin of the ^19^F peak at −104 ppm is discussed. It represents 10% of all 1D ^19^F spectral intensity. Correlating this signal to the ^31^P dimension in the 2D spectrum (−104 ppm/2.8 ppm) proves its apatite character with the preserved local arrangement. However, long-range ordering is lacking as demonstrated by the line broadening as compared to crystalline fluorapatite (Fig. 2, Table 1). We anticipate that this species is located in a boundary layer, which “heals” the mismatch of the periodic domains to get a perfect hexagonal shape of the nanoparticles. Indeed, calculation of the intensity ratio for hexagonal geometry yields the thickness of such a layer of fractions of a nm, corresponding to the size of an orthophosphate molecule. Finding an identifiable species, which we will call a boundary layer in the following, is a clue to the hexagonal arrangement of primary apatite nanocrystals observed previously using HRTEM^31^. In general, the interfacial interactions, which may be described as the glue holding the composite materials together, may comprise nonspecific (e.g. van der Waals force driven alkane-surface interactions) and specific adsorption (hydrogen bonding, coulombic ion pairing) or covalent interfacial bridging^45^. Our 2D data (Fig. 3) show that the boundary layer interacts with gelatine and water. As the CP-build up data (green line in Fig. 2c) show that the corresponding fluorine-to-proton distances are too long for the formation of covalent bonds, we suggest that specific adsorption including hydrogen bonding to the apatite PO_4_^3−^ groups is the major interfacial interaction in the present organic-inorganic nanocomposite. The ^31^P-^1^H cross-peak at 2.8 ppm/5.8 ppm (Fig. 5b) can prove the presence of such hydrogen bonded complexes as PO_4_^3−^ ··· H(w) or PO_4_^3−^ ··· H(org). The appearance of the highly ordered hydrogen bond interactions between citrate and phosphate ions in spherulites of fluorapatite has been recently demonstrated using solid-state NMR^46^.

Furthermore, two phosphorous environments different from apatite are found in the ^31^P NMR spectra (Fig. 4) at δ^31^P = 2.1 and 5.1 ppm, whose fractions are relatively small compared to the whole phosphorous content. From the 2D HETCOR data it is evident that they are not related compositionally and spatially to FAp domains and the boundary layer (Fig. 5a), but related to the proton-containing organic phase (Fig. 5b). Moreover, the ^1^H cross-correlation signal at δ^1^H = 8 ppm proves association of the negatively charged phosphate groups to the positively charged amino groups. Thus, we suggest the existence of another spatially distinguishable phase, which contains organic matrix with incorporated fluorine ions, water molecules and isolated non-apatite phosphate groups. Indeed, our 2D HETCOR NMR measurements (Figs 3 and 5b) provide clear evidence for this claim. The absence of the corresponding peaks in the ^31^P-^19^F HETCOR experiment (Fig. 5a) is explained by a low concentration of the constituents, longer distances between phosphorous and fluorine atoms in this phase or their high mobility.

In the previous simulation study on this system^47^, which addressed the mechanisms of a growth control by collagen fibers, formation of Ca_3_F motifs has been postulated. According to ref. 47 such a motif is represented by a triangle formed by calcium ions, with a fluoride ion located in the center of the triangle. The Ca···F and Ca···Ca distances range from 2.0 to 2.5 Å and 3.6 to 4.7 Å, respectively, and the angles of the triangles vary by up to 20° from the ideal value of 120°. These are incorporated into the triple helix during the embryonic stage of ion association. Such motifs represent the nucleation seeds for the formation of the oriented apatite crystal structure along the triple helix molecule. We assume here that the ^19^F signal observed at −108 ppm arise from Ca_3_F motifs, expected to occur during aggregation in/at the collagen triple helix. Indeed, its chemical shift is very close to that typical for Ca···F interactions, such as in crystalline CaF_2_^32^, although no indication for the presence of CaF_2_ has been found in the corresponding X-ray powder diffraction data (Fig. S1, Supplementary information). It is worth noting that the presence of Ca_3_F and the absence of the corresponding CaF_2_ X-ray reflections have been also found for the spherical aggregates^21^. In contrast, the formation of calcium fluoride has been found in our previous studies of fluorapatite-gelatine composites, when gelatine concentration exceeded 15 wt.%.^30^, as well as more recently, when citric acid was used as a crystal modifier for the preparation of spherical hierarchical structures of FAp^48^. We emphasize that the present work is the experimental proof of the existence of Ca_3_F and PO_4_^3−^ precursor complexes in the gelatine phase, which were not involved in the formation of apatite crystals. Alternatively, the scenario that they were expelled from the single crystalline fluorapatite domain throughout crystallization as suggested in Refs.^18^,^19^ could also be feasible.

Our experimental observations are in a good agreement with previously published molecular dynamics simulations, which provide characteristic binding positions and preferable associations of the involved ions with a gelatine molecule^47^,^49^. It has been predicted that the phosphate ions are preferentially bound outside the polypeptide strands by forming hydrogen bonds with hydroxyproline side-groups and amino groups. This gives rise to bending of the polypeptide backbone and thus leads to the observed fractal morphogenesis. The same tendency has been predicted for HPO_4_^2−^ ion aggregation. Our NMR data provide clear evidence for the existence of such phosphate groups bound to organic molecules demonstrating the corresponding NMR signals. The ^19^F signals in the range of δ^19^F = −86 to −100 ppm are attributed to the amorphous organic-related phase, which contains fluorine ions hydrogen bonded to water or/and gelatine molecules.

Finally, when all species have been identified, it is worth considering the locations and interactions of water in the nanocomposite. In this work it has been found that *(i)* the isolated water molecules are incorporated as structural defects in the crystalline FAp domains; *(ii)* bound water is present on the mineral surface, and, finally, *(iii)* mobile water is included in the amorphous organic layer, where it strongly interacts with the gelatine molecules, non-apatite phosphate groups and Ca_3_F motives. Heating of the nanocomposite to 250 °C resulted in weight loss of 0.7 wt.% (Fig. S2, Supplementary information), demonstrating that most water molecules are integrated in the nanocomposite structure. The XRD pattern after heating (Fig. S1) proves no effect on the FAp crystalline structure.

**Figure 6:**
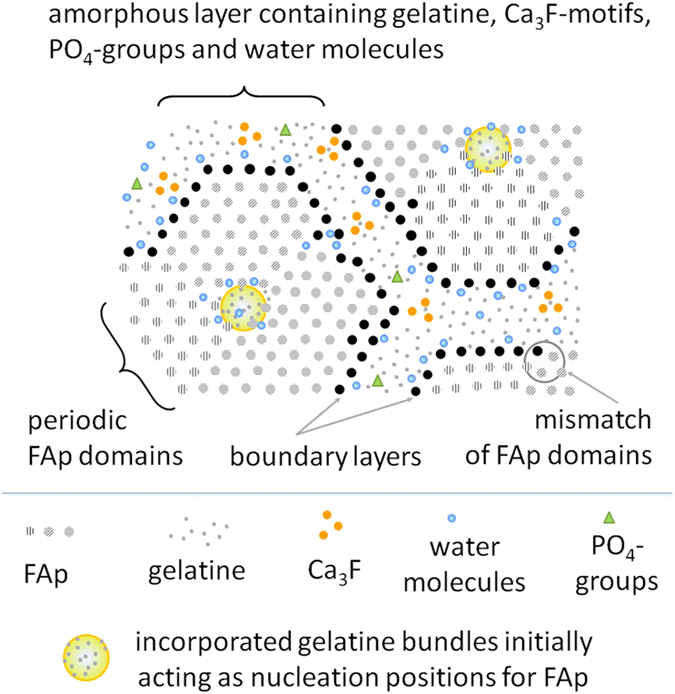
Sketch of the co-existing nano-structured mineral and organic components in the fluorapatite nanocomposite. The mineral part is represented by a mosaic arrangement of the periodic crystalline FAp domains (grey circles) surrounded by a boundary layer (black circles), which interacts with the surface water and the organic layer. The organic component incorporates Ca_3_F motifs, phosphate groups, fluorine ions and water molecules.

Based on the experimental observations in the present work, a scheme of co-existing nano-structured mineral and a superficial organic layer in the fluorapatite-gelatine nanocomposite is presented in Fig. 6. The mineral part is represented by a mosaic arrangement of the periodic fluorapatite domains (grey circles) surrounded by the boundary layer (black circles), which matches the domains to the hexagonal shape and interacts with water and the organic layer impregnated with Ca_3_F and HPO_4_^2−^/PO_4_^3−^ species.

## Conclusions

A fluorapatite-gelatine nanocomposite at the early growth stage represented by hexagonal prismatic seeds and dumbbell-like aggregates with first sights of fractal splitting has been studied. We have applied solid-state NMR spectroscopy to investigate the structure in this nanocomposite on a molecular level and provide a link between electron microscopy data, electron holography and atomistic computer simulations. Based on our results we propose a model, which demonstrates the presence of a thin boundary layer around the crystallites and of pre-nucleation clusters in the amorphous organic-containing phase. The phosphate groups in the boundary layer are involved in the hydrogen bond interactions with the organic and water molecules in the amorphous layer. Our results are in a good agreement with the theoretical predictions on the structural pre-treatment of gelatine by ion impregnation and experimental observations of an amorphous cover layer in biominerals.

## Additional Information

**How to cite this article**: Vyalikh, A. *et al.* An NMR Study of Biomimetic Fluorapatite – Gelatine Mesocrystals. *Sci. Rep.* **5**, 15797; doi: 10.1038/srep15797 (2015).

## Supplementary Material

Supplementary Information

## Figures and Tables

**Table 1:**
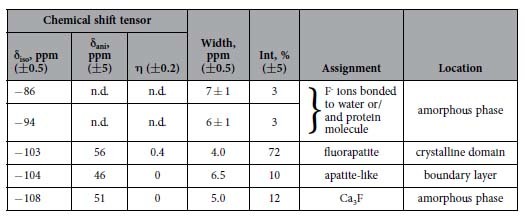
Fit parameters of ^19^F MAS NMR spectra.

Shift tensor parameters were not determined (n.d.) due to the low intensities of the corresponding spectral components.
