# Supplementary material for: An NMR Study of Biomimetic Fluorapatite – Gelatine Mesocrystals
Source: Sci Rep. 2015 Oct 30;5:15797. doi: 10.1038/srep15797 (PMC4626803; doi:10.1038/srep15797)

Supplementary information to the manuscript

An NMR Study of Biomimetic Fluorapatite – Gelatine Mesocrystals

Anastasia Vyalikh[1], Paul Simon[2], Elena Rosseeva[3], Jana Buder[2], Ulrich Scheler[4], Rüdiger Kniep[2]

[1]Institut für Experimentelle Physik, TU Bergakademie Freiberg, Leipziger Straße 23, 09596 Freiberg, Germany

[2]Max-Planck-Institut für Chemische Physik fester Stoffe, Nöthnitzer Str. 40, 01187 Dresden, Germany

[3]University of Konstanz, Physical Chemistry, POB 714, D-78457 Konstanz, Germany

[4]Leibniz-Institut für Polymerforschung Dresden e.V., Hohe Str. 6, 01069 Dresden, Germany

Figure S1. XRD patterns of fluorapatite-gelatine nanocomposite before (black line) and after (red line) heating at 250°C.

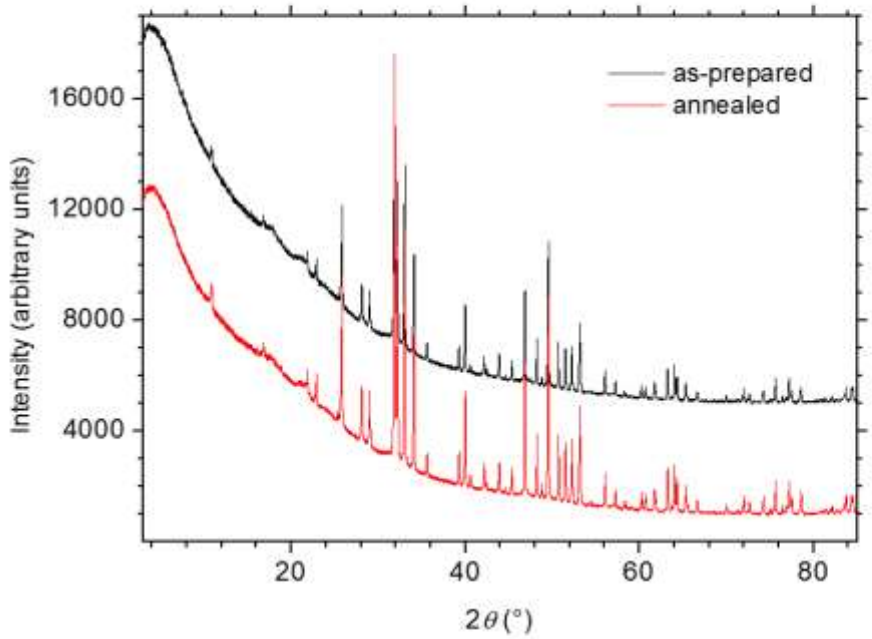

Figure S2. TGA data for fluorapatite-gelatine nanocomposite showing the weight loss of 0.7 w%.

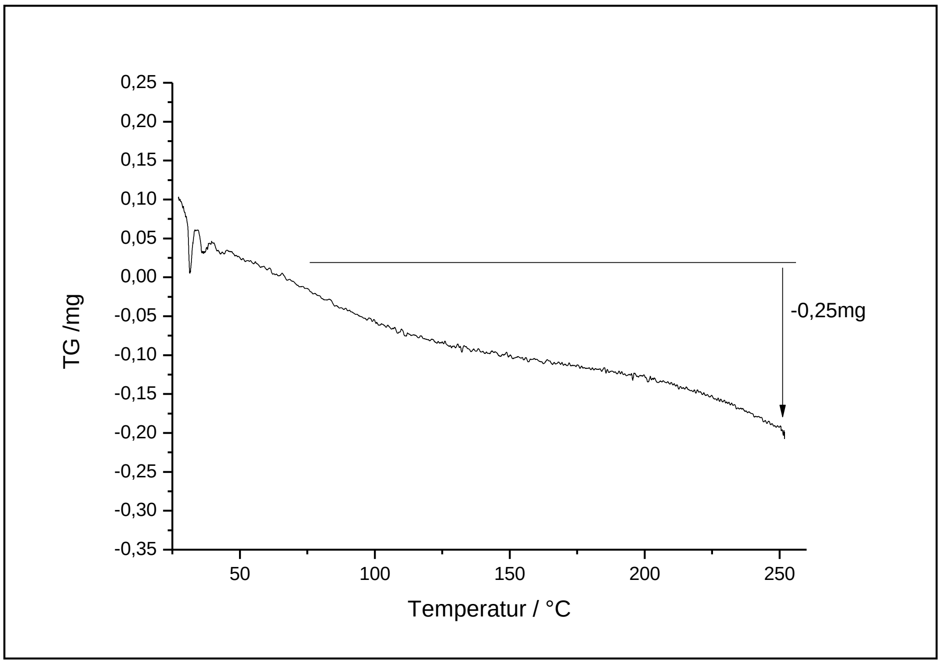

Supplement: Supplementary Information [file srep15797-s1.pdf]
